# Feeling Threatened by the War in Ukraine: A Study in Italy on Identification, Entitativity and Attitudes Toward the EU

**DOI:** 10.5964/ejop.13279

**Published:** 2025-02-28

**Authors:** Francesco La Barbera, Carmela Altamura, Roberta Riverso

**Affiliations:** 1 Department of Political Sciences, University of Naples Federico II, Naples, Italy; Nyenrode Business University, Breukelen, the Netherlands

**Keywords:** European identity, EU integration, EU enlargement, structural equation modeling, serial mediation

## Abstract

Russia’s invasion of Ukraine in February 2022 posed a practical and symbolic threat to EU citizens. Did this threat affect citizens’ identification with the EU? This was the main research question addressed in the current paper. In addition, we sought to evaluate whether the influence of perceived threat on the identification with the EU was mediated by perceived entitativity of the EU. Finally, we expected perceived threat to improve participants’ attitudes towards EU integration and enlargement, through the mediation of entitativity (Mediator 1) and identification with the EU (Mediator 2). We conducted a survey (*N* = 349, 186 females; *M*_age_ = 34.52) to assess this pattern of relations through structural equation models. Results show that perceived threat affects identification with the EU only indirectly, through the mediation of entitativity. In addition, perceived threat and entitativity have a significant indirect effect on attitude toward EU integration and attitude toward EU enlargement, yet they are directly associated only to the former. From a theoretical perspective, results are discussed in relation to previous research that shows the effect of perceived threat on identification, failing to consider the mediating role of entitativity. From a practical point of view, results may provide new insights on communication commonly used to reinforce the ingroup identity—mainly by threat-based strategies—through a re-consideration of the critical role of entitativity.

The European Union (EU) and the whole world are currently going through a time of wide crisis and transformation. The war in Ukraine has exerted a strong pressure on international relations, energy systems, and economic dynamics, challenging established balances and bringing unresolved conflicts to the fore (Deng et al., 2022; Schepers, 2022). Russia’s invasion of Ukraine in 2022 has triggered a series of consequences, ranging from reduced imports of energy commodities from Russia, to rising inflation with consequent impact on global production chains (Quintana, 2022); from reduced exports of agricultural products from Ukraine, to the global food security crisis (Ben Hassen & El Bilali, 2022), and the humanitarian crisis of refugees escaped from war (Duszczyk & Kaczmarczyk, 2022; Johannesson & Clowes, 2022; Osendarp et al., 2022; Patel & Erickson, 2022; Żuk & Żuk, 2022). In this complex scenario, the EU immediately took a stand, with a firm and shared condemnation of Russian aggression (Celi et al., 2022; Steiner et al., 2023): “Our Union as a whole has risen to the occasion […] this year, as soon as Russian troops crossed the border into Ukraine, our response was united, determined and immediate.” (von der Leyen, 2022). The economic sanctions imposed on Russia, together with the military aid provided to Ukraine, strongly characterized, in practical and symbolic terms, the EU’s position (Beauregard, 2022; Deng et al., 2022). Some preliminary empirical evidence suggests that, in EU member states, citizens show a high perceived threat in relation to the war in Ukraine, together with an overall condemnation of Russia’s invasion and positive attitudes towards sanctions against Russia (Hilbig, 2022).

In order to make a further contribution in this direction, the present study aims to explore the role of perceived threat related to Russia’s invasion on perceived entitativity of the EU and identification with EU, also considering the pattern of relations with attitudes towards EU integration and enlargement.

From a theoretical point of view, our study is guided by the social identity approach (Tajfel et al., 1971; Tajfel & Turner, 1979; Turner et al., 1987), which maintains that a part of individuals’ self-concept derives from belonging to social groups; therefore, feeling one-self as a part of the group has strong implications on its members’ attitudes and behaviors. Group membership provides attitudes and values to its members, namely a lens for interpreting the surrounding world; acting as a group member, their behaviours conform to the ingroup norms (Turner et al., 1987). Additionally, in order to achieve a positive self-concept—which depends on ingroup-outgroup social comparison—people are driven towards favoring in-group and derogating groups to which they do not belong (Brewer, 2017; Gerard & Hoyt, 1974; Tajfel, 1974).

Given the importance of group membership, the social identity approach has been used to understand several intergroup phenomena, including political events and conflicts, and applied to several contexts, such as identification with the EU. For instance, research shows that European identity and national identity can coexist because they operate at different levels of abstraction (Castano, 2004; Cinnirella, 1997). European identity fosters cooperation with European partners, predicts positive attitude towards Europe and acts of solidarity with other EU member states during crises (La Barbera, 2015; La Barbera et al., 2014; La Barbera & Cariota Ferrara, 2012; Verhaegen, 2018). This is consistent with the common ingroup identity model (CIIM; Gaertner et al., 1993; Gaertner et al., 2000), which maintains that the recategorization of different groups within a superordinate group fosters tolerance between previously divided groups, reducing perceived threat. Indeed, several studies have shown that identification with the EU improves intergroup relations between groups coming from European countries by fostering mutual tolerance (Curtis, 2014; Curtis, 2024). Drawing on social identity prospective, the uncertainty reduction hypothesis (Hogg, 2000) states that one way to reduce subjective uncertainty is through self-categorization, which is responsible for identification processes. Uncertainty “motivates people to affirm a social identity, form new groups, join pre-existing groups, and (re)construct prototypes to better resolve uncertainty” (Hogg, 2000; p. 233).

The present article is structured as follows. In the next section, we review the main literature behind our hypotheses through the lens of social identity approach. With a focus on the Ukrainian crisis, we describe the rationale underlying our model of relation between perceived threat, entitativity and identification with the EU, predicting a significant serial mediation and a significant association with attitudes towards EU integration (deepening) and enlargement (widening). In the materials and method section, we describe the sample, procedure and measures that we used to test our hypotheses. Structural equations testing our conceptual model, with the assessment of the measurement model and convergent/discriminant validity of measures, are presented in the results section. Finally, we discuss results in relation to previous studies, suggesting theoretical considerations and practical implications.

## Perceived Threat From Russian Invasion and Identification With the EU

There are several reasons which might explain why the perception of threat related to the Ukrainian crisis is relevant among EU citizens, even if not directly involved in war. First, the sanctions EU imposed on Russia have had large negative consequences in the EU member states and in citizens’ everyday life, such as the rising of prices of energy, raw materials and consumer goods (Berner et al., 2022; Rühl, 2022). Therefore, even if not directly involved in the war and battlefields, EU’s citizens are bearing war-related hardships (Guenette et al., 2022). In addition, some governments, such as Italy’s, have implemented measures to curb the energy consumption. This may well pose a threat to citizens’ well-being and lifestyle (Barchielli et al., 2022). Second, the possibility of the conflict escalating into the use of nuclear weapons depicts a life-threatening scenario also for European countries not directly involved in the war (Bollfrass & Herzog, 2022). Third, geographical proximity of EU countries with Ukraine and Russia should also be considered, because it is a variable thought to increase dramatically the sense of threat (Gehring, 2019). Finally, it is important to consider the threat that Russia poses on EU values. EU has traditionally proposed its foundation in values of human rights, human dignity and freedom, and EU is widely seen as a democratic and humanitarian international agent (Giumelli et al., 2021; Steiner et al., 2023). Hence, Russia’s invasion of Ukraine poses a symbolic threat to basic EU’s values, as it has been explicitly stated several times by the highest authorities of the EU institutions: “This is not only a war unleashed by Russia against Ukraine. This is a war on our energy, a war on our economy, a war on our values and a war on our future. This is about autocracy against democracy.” (von der Leyen, 2022, p. 3). This might also increase EU citizens’ perceived threat; in addition, threatening the ingroup values may increase the effect of identification on ingroup favoritism and outgroup derogation (Bianco et al., 2022; Voci, 2006).

Our first research question—on which there is currently no specific empirical evidence—is whether this sense of perceived threat is influencing the strength of citizens’ identification with the EU, which has often been seen as weak and problematic (Carl et al., 2019; La Barbera & Ajzen, 2020).

Research in social and experimental psychology has shown that perceived threat increases individuals’ identification with the ingroup (Festinger, 1950; Hogg, 2000; Smeekes & Verkuyten, 2013; Tajfel & Turner, 1979), which acts as a buffer against individual perceived vulnerability. Hence, from a concrete point of view, EU’s citizens may perceive the EU as a powerful alliance and a source of safety, stronger and more reliable than the single member states (Gehring, 2019). Importantly, facing a common threat, EU citizens may categorize themselves as “us” in contrast to “them”, increasing their identification with the superordinate common ingroup (Gaertner & Dovidio, 2014). This would be consistent with the social identity approach, according to which the perceived threat can lead people to feel closer to the ingroup and increase aggression towards the outgroup (Tajfel & Turner, 1979; Vergani, 2018), and with previous studies related to Ukrainian crisis that have shown a “rally effect” around the EU flag (Steiner et al., 2023) and positive effects of the war in Ukraine on the EU integration process (Fiott, 2023; Håkansson, 2024). In addition, this line of reasoning is somewhat supported by considerations drawn in relation to Russia’s invasion of Crimea in 2014. In a secondary analysis of Eurobarometer data, Gehring (2019) found a significant increase of EU identification in countries theoretically assumed to be more threatened by Russia’s aggressive behavior (Estonia and Latvia, countries bordering Russia directly). This increased identification with EU was accompanied by increased trust in EU institutions and greater support for EU policies at a central level. Steiner and colleagues (2023) also report an improvement of EU identification and attitudes towards the EU. These studies did not provide direct measurement of perceived threat; nevertheless, they support us in expecting that EU citizens’ level of perceived threat from the Russian aggression in Ukraine of February 2022 should be significantly and positively associated with identification with EU.

Second, we sought to explore whether this effect of perceived threat on identification with EU was mediated by perceived entitativity of the EU. Entitativity has been described as the psychological existence of a group (Castano, Sacchi & Gries, 2003)—that is, the extent to which the group is perceived as a real entity—and it has been linked to beliefs about the group members’ sharing of common values and common past, having things in common and being similar on personal traits and characteristics (Agadullina & Lovakov, 2018). Highly entitative groups are perceived as a whole, homogeneous and cohesive entity (Agadullina & Lovakov, 2018; Yzerbyt et al., 2000). Research shows that the ingroup entitativity strengthen ingroup identification (Sacchi et al., 2009), and perceived entitativity of the EU has been found to increase individuals’ levels of identification with EU (Castano, 2004). Research has also shown that individuals identify more strongly with an ingroup when they are in a condition of uncertainty and the group is highly entitative (Hogg et al., 2007), and perceived threat may exert a direct effect on entitativity (Jutzi et al., 2020). It is reasonable to think that Russia’s invasion of Ukraine has increased EU’s entitativity; for example, creating the perception of common fate between European citizens (Castano, Yzerbyt & Bourguignon, 2003). In addition, the threat related to Russia’s invasion of Ukraine may increase EU’s entitativity by enhancing the importance citizens assign to commonality rather than differences between European peoples, because external threats have been found to reduce the perceptions of dissimilarities (Vezzali et al., 2015) and enhance the perceived similarity of ingroup members (Rothgerber, 1997). Therefore, Russia’s invasion of Ukraine might have both a direct effect on identification and an indirect effect through enhancing EU’s entitativity.

## Threat, Identification, and EU-Related Attitudes

Research has shown that the strength of identification with the EU is a predictor of a number of very important variables (La Barbera, 2015; La Barbera et al., 2014), such as attitudes in favor of EU integration (EU deepening) and EU enlargement (EU widening). Despite the EU enlargement has often been considered as a part of EU integration process, they are two different concepts because the support for EU integration is not always accompanied by support for enlargement and *vice versa* (Karp & Bowler, 2006). Attitude toward EU deepening has been defined as the attitude citizens hold toward the increase of EU’s power in political and economic decision-making, even in substitution of national sovereignty (Castano, 2004; La Barbera, 2015). This attitude is especially important, because EU deepening is an ongoing process of progressive integration and centralization of power aimed at an ever-closer union; although the most part of international issues require cooperation and trans-national solutions that transcends national boundaries (Buchan et al., 2011), EU integration process has been in many occasions contrasted by public opinion, citizens at referenda, and political parties in different member states (La Barbera & Ajzen, 2020). From a social identity perspective, European identity is a pre-condition for legitimacy and citizens supporting EU integration (Castano, 2004; Fuchs & Schlenker, 2006). This is in line with research showing that citizens’ identification and attachment with their own nation and with EU influences support for EU integration process (Herrmann et al., 2004; La Barbera, 2015) and promote cooperation between citizens from different European countries (La Barbera et al., 2014; La Barbera & Cariota Ferrara, 2012).

Although initially the EU enlargement literature was centered on the elites’ politics and preferences, exploring public attitudes towards enlargement has been considered essential to ensure stability in Europe (Maier & Rittberger, 2008). The enlargement of the EU is related to the issue of demarcation between who is a group member and who is not, and it has stimulated a wide debate among scholars (Maier & Rittberger, 2008; McLaren, 2007; Thomassen, 2008; Vetik et al., 2006; Zielonka, 2007). Attitude toward EU enlargement has been found to be positively correlated with attitude toward EU integration and identification with EU (Boomgaarden et al., 2011; de Vreese & Boomgaarden, 2006; Fuchs & Schlenker, 2006). Previous research on the point also found evidence of a causal effect of identification with EU on attitude towards EU widening (La Barbera, 2015).

The EU’s enlargement policy has navigated through various phases and geopolitical contexts to build a united and prosperous Europe ensuring stability and democracy in all member countries (Schimmelfennig & Kakhishvili, 2024). This notwithstanding, Russia’s invasion of Ukraine created an unprecedented situation as regards EU enlargement. On 28 February 2022, the government of Ukraine signed an application for EU membership, asking for immediate accession; three days later, Georgia and Moldova submitted similar applications. EU leaders decided to grant Ukraine and Moldova the status of candidate country at the June European Council. This unprecedented immediate EU’s positive response to an application for EU membership has been publicly defined as a “political gesture” by French President Emmanuel Macron, which would not have been possible without the war in Ukraine and the fight of its people “to defend our values, their sovereignty, their territorial integrity” (Sapir, 2022). Therefore, EU widening might be at a turning point, and the reaction of member states’ citizens toward this unprecedented enlargement policy is largely unknown.

## Aims and Hypotheses

In the current study, we aimed to test whether the pattern of relations deriving from the theoretical considerations outlined above was supported by data. We evaluated the relation between participants’ perceived threat related to Russia’s invasion of Ukraine and their strength of identification with the EU. We also tested whether this relation was mediated by perceived entitativity of the EU. In addition, we sought to explore the pattern of relations between perceived threat, perceived entitativity of the EU, and EU identification, with EU-related attitudes, namely the attitude toward EU integration (EU deepening) and enlargement (EU widening).

**Figure 1:**
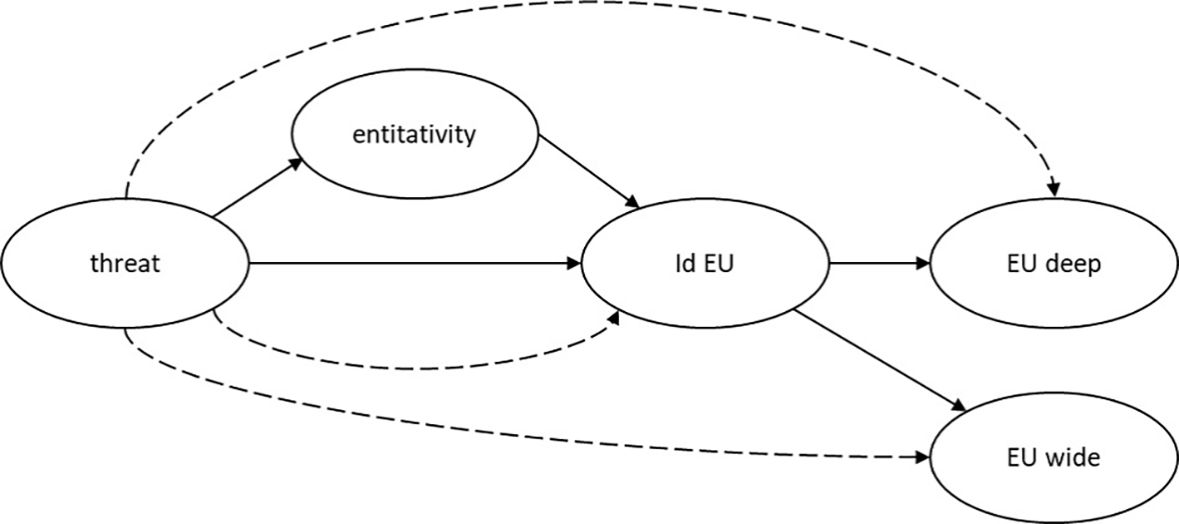
Conceptual Model of Relations Between Perceived Threat, Entitativity, Identification With EU and Attitudes Toward EU Deepening and Widening *Note.* The figure depicts the conceptual model of the effect of perceived threat (threat) on attitude towards EU integration (EU deep) and EU enlargement (EU wide) *via* entitativity and identification with EU (Id EU). Hypothesized direct effects are represented with arrows; indirect effects are depicted by dotted lines.

Specifically, we hypothesized to find a significant association of perceived threat with identification with EU (**H1**) as well as between identification with EU and attitude towards EU deepening (**H2**) and EU widening (**H3**). In addition, we expected to find a significant indirect effect of perceived threat on attitude toward EU deepening (**H4**) and EU widening (**H5**) *via* entitativity (Mediator 1) and identification with EU (Mediator 2). We also expected the effect of perceived threat on identification to be mediated by perceived entitativity of the EU (**H6**). Perceived threat (**H7**) and entitativity (**H8**) are expected to have significant association with attitude towards EU deepening, whereas their direct association with attitude towards EU enlargement—drawing on the considerations outlined above—was more difficult to predict and has been evaluated in a more explorative fashion. Figure 1 summarizes the conceptual model.

## Method

### Participants

A convenience sample of 350 Italian participants was recruited in March 2022. Participants were recruited through an online snowball sampling, sharing the link to the online questionnaire on social networks (e.g., Facebook) and asking people to further share the link with friends and acquaintances on their own social network page and/or through instant messaging apps (e.g., WhatsApp). One respondent failed to complete half of the questionnaire, thus was removed from final dataset (see La Barbera, 2025), which consists of 349 individuals, 186 of whom identified themselves as females, 124 as males, 1 as non-binary and the remaining 38 preferred not to communicate their gender. The mean age of the sample was 34.52 (*SD*_age_ = 14.58), ranging from 18 to 87. Slightly less than half participants (170) hold a university degree and 137 completed high school.

### Procedure

Participants volunteered for the study and received no compensation for their participation. They were assured of anonymity and informed that they were free to discontinue participation at any time without penalty. After giving their consent, they completed an online questionnaire containing the measures described below. The items employed a 7-point response format and were presented in non-thematic order. The full list of items, with descriptive statistics, is provided in Table A1 in the Appendix.

### Measures

#### Perceived Threat

Five items were used to measure the individuals’ perceived threat from Ukrainian crisis. Drawing on previous research (Riverso et al., 2022), items made reference to general concern (*e.g*., *In general, how concerned are you about the war in Ukraine?*), perceived probability and seriousness of negative consequences, direct and indirect perceived risks. Participants answered on a 7-point response scale. Responses were averaged, with higher values indicating higher perceived threat (Cronbach’s α = .85).

#### Perceived Entitativity of the EU

Drawing on the work by Agadullina and Lovakov (2018), we used three items for measuring participants’ perceived entitativity of the EU. The items tapped into perceived similarity *(e.g., European citizens are very similar to each other)*, commonalities, and shared past of the group members. Participants answer on a scale from 1 (Completely disagree) to 7 (Completely agree). A composite measure was computed by averaging the scores (Cronbach’s α = .83). Higher values indicate a higher perceived entitativity of the EU.

#### Identification With EU

Participants’ identification with EU was measured using the four-item identification scale by Spears and colleagues (1997), in the Italian version used by La Barbera (2015) for measuring participants’ strength of identification with EU. Participants indicated their agreement or disagreement for each statement (*e. g., I think about myself as a European citizen*) on a scale from 1 to 7. A composite measure was computed by averaging the scores (Cronbach’s α = .87). Higher values indicate a higher level of identification with EU.

##### Attitude Towards EU Deepening

Participants’ attitude toward the integration of the EU (EU deepening) was measured by means of the five-item scale by La Barbera (2015). Participants indicated their agreement or disagreement for each statement (*e. g., I see positively a more immediate action in applying European norms*) on a scale from 1 to 7. A composite measure was computed by averaging the scores (Cronbach’s α = .86). Higher values indicate more positive attitude toward EU deepening.

##### Attitude Towards EU Widening

Participants’ attitude toward the enlargement of the EU (EU widening) was measured by means of the five-item scale by La Barbera (2015). Participants indicated their agreement for each statement (*e. g., I see positively the enlargement of the EU beyond the countries that have joined from the beginning*) on a scale from 1 (Completely disagree) to 7 (Completely agree). A composite measure was computed by averaging the scores (Cronbach’s α = .92). Higher values indicate more positive attitude toward EU widening.

### Statistical Analysis

As regards the main objective of the study, the *a priori* power analysis—ran by the software G-Power 3.1—indicated a sample of 287 participants in order to detect a small effect (*f^2^* = .03) with 1 – β = .90 and α = .05.

Descriptive statistics, correlations between measures, t-tests and reliability tests have been conducted by the software SPSS 22 (IBM). The software Stata 15 (StataCorp LLC) has been employed in order to perform structural equation modeling, including the assessment of the measurement model and convergent/discriminant validity of measures. The level of significance was set at *p* < .05.

## Results

The average scores and intercorrelations of the study variables are shown in Table 1. Perceived threat and identification with EU were significantly over the scale mid-point, *t*(348) = 16.68, *p* < .001 and *t*(348) = 5.43, *p* < .001, whereas entitativity was not, *t*(348) = 1.25, *p* > .20. Attitude towards EU deepening was not different from the scale mid-point, *t*(348) = 1.85, *p* > .05, whereas the average score of attitude towards EU enlargement was significantly different, *t*(348) = 6.25, *p* < .01. The correlations between all the study variables proved statistically significant. Noticeably, the associations of perceived threat with entitativity, EU identification and EU attitudes were weak, whereas the intercorrelations among entitativity, EU identification and EU attitudes were moderate to large.

**Table 1:** Means, Standard Deviations, and Intercorrelations of the Study Variables

Construct	1	2	3	4	5
1. Threat	5.12 (1.25)				
2. Entitativity	.167*	3.90 (1.47)			
3. Ideu	.183*	.720**	4.44 (1.51)		
4. Deep	.209**	.472**	.494**	4.16 (1.65)	
5. Wide	.212**	.449**	.496**	.541**	4.52(1.54)

*Note.* Diagonal cells show means (*SD* in parentheses). Threat = perceived threat; Entitativity = Perceived entitativity of the EU; Ideu = Identification with EU; Deep = Attitude toward EU deepening; Wide = attitude toward the enlargement of EU.

*p < .01. **p < .001.

A SEM was run with 22 items reflecting five correlated latent factors: perceived threat (threat), perceived entitativity (entitativity), identification with EU (ideu), attitude towards EU deepening (deep), attitude towards EU widening (wide). The measurement model fit-to-data was sufficient (Hu & Bentler, 1999): TLI = .920; CFI = .930; RMSEA = .070. The full list of items with CFA loadings and descriptive statistics is provided in Table A1 in the Appendix.

The item’s standardized loadings ranged from .58 to .92, *p* < .001. The AVE was above .50 for all latent constructs and always exceeded the squared correlations between them (see Table 2), thus indicating convergent and discriminant validity (Fornell & Larcker, 1981).

**Table 2:** Convergent and Discriminant Validity Assessment for the Latent Constructs

Construct	1	2	3	4	5
1. Threat	.543				
2. Ideu	.047	.633			
3. Deep	.061	.306	.554		
4. Entitativity	.032	.576	.266	.674	
5. Wide	.043	.274	.327	.179	.706

*Note.* Diagonals are average variance extracted (AVE) by each latent construct. Lower diagonals are squared correlation coefficients.

In order to assess the presence of a significant common method bias, we also run an alternative model with one latent factor reflected in all 22 items. Common method bias is present if the one factor model fits the data as well as the hypothesized model (Korsgaard & Roberson, 1995). The fit-to-data of the one-factor model was unsatisfactory, TLI = .477; CFI = .526; RMSEA = .177.

In line with our expectations, the association between threat and entitativity was significant, β = .33, *z* = 2.97, *p* < .01, as it was the association between entitativity and ideu, β = .81, *z* = 14.72, *p* < .001. In addition, ideu had a significant direct effect either on deep, β = .36, *z* = 4.17, *p* < .001, and on wide, β = .46, *z* = 5.32, *p* < .001. The direct effect of threat on ideu was not significant, β = .17, *z* = 1.91, *p* = .056, whereas the total effect of threat on ideu was significant, β = .44, *z* = 3.59, *p* < .001. In addition, threat showed a significant direct effect on deep, β = .24, *z* = 2.39, *p* < .05, yet not on wide, β = .19, *z* = 1.89, *p* = .059. Similarly, entitativity exerted a significant effect on deep, β = .20, *z* = 2.22, *p* < .05, but not on wide, β = .05, *z* < 1.

As regards indirect effects, the double-mediation path from threat to deep *via* entitativity (Mediator 1) and ideu (Mediator 2) was significant, β = .22, *z* = 3.39, *p* < .001. The indirect effect of threat on wide *via* entitativity (Mediator 1) and ideu (Mediator 2) was significant as well, β = .22, *z* = 3.43, *p* = .001. Finally, we found a significant indirect effect of threat on ideu *via* entitativity, β = .27, *z* = 2.93, *p* < .01; a significant indirect effect of entitativity on deep, β = .29, *z* = 4.09, *p* < .001, and wide, β = .38, *z* = 5.11, *p* < .001, *via* ideu.

**Figure 2:**
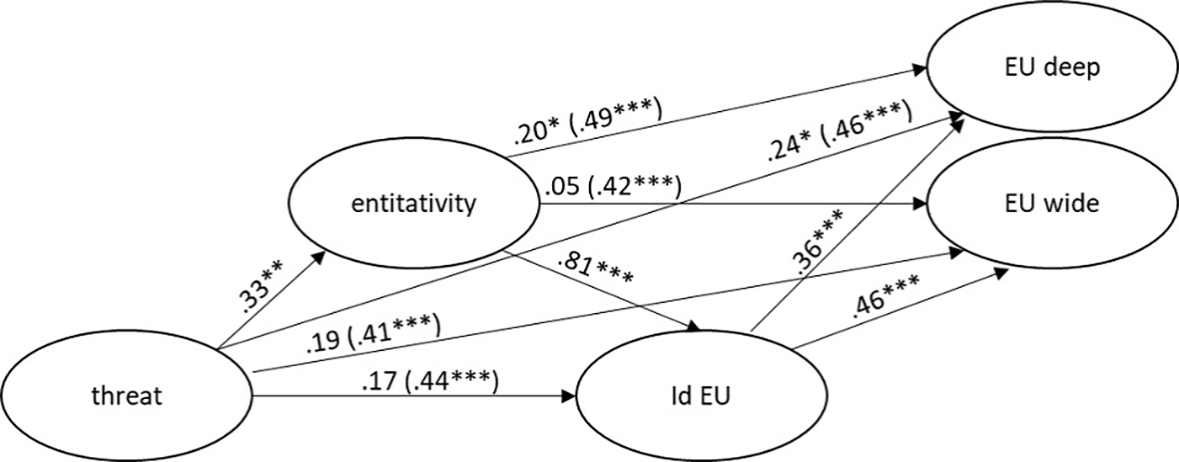
Structural Equation Model of the Effect of Perceived Threat on Attitude Towards EU Integration and Enlargement via Entitativity and Identification With EU *Note.* Structural equation model of the effect of perceived threat (threat) on attitude towards EU integration (EU deep) and attitude towards EU enlargement (EU wide), via entitativity and identification with EU (id EU). Direct effects are shown on arrows (total effects in parentheses). *p < .05. **p < .01. ***p < .001.

Total and direct effects are shown in Figure 2.

## Discussion

Results provide support to the hypothesized indirect effect of perceived threat related to Russia’s invasion of Ukraine on attitudes toward a further integration of the EU (**H4**) and EU widening as well (**H5**), through entitativity and citizens’ identification with EU. EU identity is directly associated with both the EU-related attitudes (**H2** and **H3**). Importantly, the direct influence of perceived threat on EU identification is not significant (in contrast with **H1**), and the correlation between these two variables is significant but relatively small. Perceived threat has been found to influence indirectly citizens’ identification with the EU, through entitativity (**H6**). Perceived threat and entitativity have a significant direct effect on attitude towards EU integration (**H7** and **H8**); they do not exert any direct influence on attitude towards EU enlargement, instead.

The notion that large and heterogeneous political entities such as EU are kept together and reinforced by the fear of an external threat is very common in the history of human thought (Montesquieu, 1777; Simmel, 2010; Steiner et al., 2023). Perceived threat deriving from Russia’s invasion of Ukraine might influence identification with EU directly, yet also indirectly, through the raising of EU’s perceived entitativity. Our findings support the latter scenario. Noticeably, if entitativity was the direct antecedent of EU identification, which mediates the effect of perceived threat, the traditional idea of conflict and threat as necessary conditions for identity would be weakened. Perceived threat has been shown to be only one possible antecedent of ingroup entitativity, which can be enhanced also by perception of common fate, perceived similarity, communication and symbols (Callahan & Ledgerwood, 2016; Deng et al., 2022). From a practical point of view, this could mean that political efforts to promote EU identity may be directed toward strengthening the EU’s entitativity, rather than toward the identification of common enemies.

Our findings confirm and expand previous research showing that individuals identify more strongly with an ingroup when they are uncertain *and* the group is highly entitative (Hogg et al., 2007). Our findings are also in the path of previous research suggesting that entitativity may play a key role in intra- and intergroup relations: Ingroup entitativity may foster perceived groups’ efficacy, whereas not necessarily determining outgroup derogation (Yzerbyt et al., 2000).

Individuals identify with their ingroup and tend to defend its values, because this enhances their sense of self-continuity (Smeekes & Verkuyten, 2013). Hence, the symbolic threat that Russia’s invasion represents—which appears amplified in the narratives of EU’s highest representatives—may well strengthen citizens’ identification with the EU. Nevertheless, at least in the case of the EU, our findings show that this influence is mediated by entitativity, and entitativity—not perceived threat—is the direct antecedent of EU identity. This seems original and different from the widespread idea that perceived threat is the direct antecedent of ingroup identification (Brewer, 2011; Vergani, 2018). However, our findings are not necessarily incompatible with results of previous research on which this idea relies upon. First, research showing a significant direct link between perceived threat and ingroup identification did not consider/test entitativity as a mediator (Giles & Evans, 1985; Greenaway & Cruwys, 2019); without considering the role of entitativity, also our studies would have shown a significant (total) effect of threat on identification. In this sense, the current research is not in contrast with previous psychological-social literature on threat-identity relation, yet expands it considering the mediating role of entitativity. Second, our study suggests that entitativity fully mediates the effect of perceived threat on identification in relation to the EU, which is a supranational/supraordinate group with its own characteristics. As such, the full or partial mediation of entitativity cannot be taken for granted in relation to other groups, categories, and contexts. Future research could fruitfully investigate whether this mediation is also relevant in relation to different context and groups, or whether it is specific to European identity.

Our findings show that perceived threat exerts a significant indirect effect—via identification with EU—on attitudes towards EU’s deepening and widening. Perceived threat also directly affects citizens’ attitude towards EU’s deepening, yet it has no direct effect on their attitude towards EU’s enlargement. This different association of perceived threat to those two attitudes seems in line with research showing that, when individuals feel threatened, they not only tend to identify more strongly with their group, but also tend to operate a cognitive closure to the outside (Hogg & Adelman, 2013; Kruglanski et al., 2006). This closure involves the rejection of any change and the desire to maintain a stable and immutable situation, predictable and familiar (Bianco et al., 2022; Federico et al., 2013).

In a similar fashion, entitativity is directly associated with attitude towards EU deepening, whereas the direct association with attitude towards EU widening is not significant. This result could be interpreted in line with research on essence-based vs. agency-based perceived entitativity (Brewer et al., 2004): the entitativity of a group may be based on perceive common properties or in terms of their goals and actions. In this study, we measured entitativity with items that mostly address the essence-based entitativity. However, previous research suggests that essence-based entitativity—compared to agency-based entitativity—may be less correlated to the attitude towards EU enlargement (La Barbera, 2015). Therefore, future developments of the current research may deepen the role of entitativity in the pattern of relations between perceived threat, EU identification and EU-related attitudes, distinguishing between essence-based and agency based entitativity.

In sum, the present research extends the literature on the relation between European social identity, threat and entitativity; although previous research have shown a significant effect of threat and entitativity on identification, the present study provides specific evidence related to the EU, as well as the threat deriving from the war in Ukraine and suggests that, at least in the case of the EU, threat is not the direct antecedent of group identification, because its influence is (totally) mediated by entitativity. In addition, the present study extends the literature on attitudes towards EU deepening and widening, showing that the perceived threat fosters attitude towards EU’s deepening and widening whereas entitativity influences only attitudes towards integration; however, further research is needed on this point. Therefore, our findings suggest that the perception of the EU as a cohesive and united entity is crucial to strengthen the sense of belonging to the EU as well as more positive attitudes toward EU integration and enlargement. Politics and governments might work/focus on two main aspects: awareness of the role of the EU in citizens’ life and on the perception of a united Europe. Strengthening the EU’s social policy and reducing disparities between EU countries could promote trust in the EU and shared sense of belonging, increasing awareness of the role of the EU in citizens’ lives as well. Drawing on previous research about different contents of European identity (La Barbera, 2015), the EU’s institutional policies and communication might also try to strengthen entitativity focusing on common goals and directions, rather than on characteristics linking people through (the construction/representation of) a common history.

Our studies present several limitations that need to be acknowledged. First and foremost, the research work has been conducted with a convenience sample and all participants were from Italy. In addition, the education level of our sample is higher than the average population in both Italy and the EU, whereas the mean age is lower than the average age of Italians (*M* = 46.4) as measured in 2022 by the Italian Institute of Statistics (ISTAT, 2023). The online snowball sampling allowed us to obtain an adequate number of participants in a relatively brief amount of time, as we were interested in measuring individuals’ attitudes and perceived threat potentially correlated to Russia’s invasion soon after the event. Nevertheless, the characteristics of our sample raise concerns about the generalizability of results. Overall, the insights provided in the current article should need to be supported by further research, conducted on large and multi-national samples. It would be interesting to test the pattern of relations proposed in the current study—specially the effect of threat on EU identification fully mediated by entitativity—in different countries, such as Northern and Southern European member states, early and new member states, states with different distance from Russia, and so forth.

The correlational nature of the study poses a limitation to the strength of causal assumptions implied in the conceptual model proposed. This notwithstanding, the context of a historical situation in which Europeans are exposed to real threatening scenarios was a precious opportunity to gain knowledge on the relations between citizens’ perceived threat, perceived entitativity of the EU, identification with the EU, and attitudes towards EU integration and enlargement. Experimental assessment of this pattern of relations might of course help in shedding light further on the topics addressed in the paper, especially regarding the assumptions of causality between the study variables.

## Supplementary Materials

For this article, the following Supplementary Materials are available:

- Data. (La Barbera, 2025)
- Code. (La Barbera, 2025)
- Supplementary materials. (La Barbera, 2025)

## Data Availability

For this article, data, codebook and materials are available at La Barbera (2025).

## Appendix

### Table A1

**Table:**

Items (and latent constructs)	CFA loading	*M* (*SD*)
(Perceived threat)		
In general, how concerned are you about the war in Ukraine?	.59	5.82 (1.40)
How likely do you think it is that the war in Ukraine will negatively affect your and/or your loved ones’ lives?	.83	5.45 (1.60)
How serious do you think the possible negative repercussions of the war in Ukraine are likely to be on your and/or your loved ones’ lives?	.88	5.25 (1.43)
I believe that in the coming months the war in Ukraine may pose a direct danger and put my and/or my loved ones’ safety at risk.	.64	4.31 (1.81)
I believe that in the coming months the war in Ukraine may have negative consequences on my level of well-being.	.70	4.75 (1.68)
(Entitativity)		
European citizens share a common past.	.59	4.29 (1.83)
European citizens have many things in common with each other.	.92	3.86 (1.64)
European citizens are very similar to each other.	.90	3.56 (1.64)
(Identification with EU)		
I think about myself as a European citizen.	.88	4.17 (1.88)
I feel I am deeply tied to other European citizens.	.60	4.23 (1.83)
Overall, I am glad to be a European citizen.	.75	5.50 (1.57)
Being a European citizen fully represents me.	.91	3.86 (1.86)
(Attitude towards EU deepening)		
EU should be a single country.	.68	4.13 (2.25)
I am in favor of a centralized management scheme of the European economy to replace the economic powers of individual states.	.77	3.98 (2.07)
I see positively a more immediate action in applying European norms.	.70	4.55 (1.78)
I would be in favor of the creation of one single European army to replace national armies.	.76	4.17 (2.12)
I would be in favor of giving a greater political power to the EU, greater than the power of each single country.	.80	3.96 (2.09)
(Attitude towards EU widening)		
I see positively the enlargement of the EU beyond the countries that have joined from the beginning.	.92	4.69 (1.75)
I think that EU enlargement represents an opportunity for all of us.	.92	4.71 (1.76)
Italy will experience more advantages than disadvantages from the enlargement of the EU.	.77	4.42 (1.77)
The enlargement of the EU is important for the future of the EU.	.81	4.54 (1.77)
For me personally, the enlargement brings more advantages than disadvantages.	.76	4.23 (1.78)
